# Forms of uncertainty reduction: decision, valuation, and contest

**DOI:** 10.1007/s11186-018-9311-0

**Published:** 2018-03-11

**Authors:** Patrik Aspers

**Affiliations:** Department of Sociology, Uppsala University, Box 624, 751 26 Uppsala, Sweden

**Keywords:** Competition, Game, Market, Ranking, Status, Value

## Abstract

Uncertainty is an intriguing aspect of social life. Uncertainty is epistemic, future-oriented, and implies that we can neither predict nor foresee what will happen when acting. In cases in which no institutionalized certainty about future states exists, or can be generated, judgment is needed. This article presents the forms by which uncertainty is reduced as a result of judgments made about different alternatives in a process involving several actors. This type of uncertainty may exist, for example, about which artist is the best, which offer in the market is more valuable, which football team is better than all the rest, or which research proposal will get a grant. The result of different forms of uncertainty reduction is increased certainty concerning alternatives in relation to one another, such as good and bad, rank lists, scores, quality assessment, and “winner and losers.” Based on the result, uncertainty is reduced and action is facilitated. The forms are structural and comprise roles; may be legitimate in a smaller or larger domain; and may exist in all spheres of life, as exemplified in sports competitions, in labor markets, and in the ranking of universities. Three forms of uncertainty reduction based on judgment are identified in this article: (1) decision, made by an authority; (2) valuation, by means of which order arises as a result of actors ascribing values; and (3) contest, by which an order is the result of direct struggle.

Uncertainty is an intriguing but essential aspect of social life. Although total certainty would probably be unbearable, we are often plagued by uncertainty. Uncertainty is epistemic, future-oriented, and refers to a lack of knowledge concerning how to act with predictable outcomes. Uncertainty means that we cannot predict or foresee what will happen when acting or not acting (Beckert 1996, pp. 3–4; Beckert 2016; Keynes 1973; Knight 1921).

The future is unknown by definition, but over time human ingenuity and established practices have given us a stock of institutionalized knowledge about matters that reduce uncertainty. This requires involvement of several actors and some kind of publicness (Berger and Luckmann 1991). Law and scientific work are paradigmatic examples of establishing certainty with regard to existing states and correlated predictability between states. Also social conventions, e.g., norms and practices, facilitate decision making.

By employing our current knowledge and the combination of states of what I call institutionalized certainty we can not only predict, but also fashion, the future. This is the case when we use pesticides to protect plants from various diseases, but they may also pollute the water. These predictions are based on statements of institutionalized certainty. We have thereby, for example, been able to leave the life of European Middle Ages, i.e., at the time humans lived with no security and when one could not calculate for the future (Elias 1969, p. 277) behind us in many parts of the world. By ordering the world, either in the form of a decided order, for example setting laws, standards, and rules, or out of mutual adjustment by interacting actors who develop conventions, uncertainty is reduced. This knowledge, however, is only “institutionalized” in a given context and not true in an absolute sense (Heidegger 1988; Quine 1961).^1^ It may even be “wrong.” There are, for example, many rules of thumb, as institutionalized knowledge, that people make use of, but that may be incorrect (Kahneman and Tversky 1979; Tversky and Kahneman 1974). It is, nonetheless by and large taken for granted and for the time being not put in question. Institutional knowledge refers to codified praxis, such as legal system, standards to facilitate coordination (such as the metric standard), and states generated by science (as in scientific propositions backed by evidence). Much of this part of the lifeworld is that upon which more explicit and codified institutions rest (Berger and Luckmann 1991; Husserl 1970). The line between what we “know” and not know has changed over time due to the growth of an extended body of knowledge (Pareto 1935).

In many cases uncertainty refers to states that are not institutionalized. Some of these states cannot even be “known,” which means that they have not yet been, or cannot be, reduced to “laws,” or standards. In these cases judgment by other means is needed. Judgment may, for example, be used to find out what is a good action in, for example, a moral, aesthetic sense; when one is uncertain about the quality of the paper one has just finished; for identifying the best candidate for a job; or to identify the best sailor in the Laser boat class. Such judgements, if reached in social processes, may reduce uncertainty.

By which means is uncertainty reduced by result of judgments? This article analyzes forms (cf. Simmel 1923, p. 47) of uncertainty reduction in cases when no institutionalized certainty exists. It presents and analyses three forms of uncertainty reduction and looks at the questions to which they give rise. The upshot of applying these forms is to increase the certainty of alternatives in relation to one another, in terms of rank lists, scores, quality assessments, and “winners and losers.” By their means, uncertainty concerning—in the widest sense—what is good and bad or what has worth and what does not, is reduced. *Forms* of reduction refer here to the social structure of roles that reduce uncertainty. This article studies forms of uncertainty reduction and its public implications that affect several actors. Furthermore, the outcome of the forms is not institutionalized. Based on the “judgments” of these forms, actions are facilitated.

The forms may be legitimate in a smaller or larger domain, existing in all spheres of life—for example, in sports competitions such as the US Open in tennis, as well as in labor markets, and when universities are ranked. Deciding what car to buy, what film to see, or which of the 500 academic papers on trust on the desk in front of us we should read requires reduction of uncertainty. The concrete output may be a statement of which actions are good, a rank list of job applicants, or a winner in a regatta.

This text makes a contribution, above all, to the field of valuation studies. The problem of what are good and bad, in the widest sense, is the theme in this nascent field. Valuations have become common in contemporary society, but have always been central in social life: “man designated himself as the creature that measures values, evaluates and measures, as the ‘valuating animal as such,’” declared Nietzsche (1994, p. 70). The centrality of valuation, both as social phenomena and a topic of academic interest, is acknowledged by researchers. There are many empirical studies, as shown in two comprehensive overviews of the field (Lamont 2012; Zuckerman 2012), and the related work by Fourcade (2016) on ordinalization. Little attention is paid, however, to the ways in which reduction of uncertainty is carried out, and no attention at all to the discussion of more generally existing forms that might be observed. Markets are the obvious case of a form, but markets are often discussed in their own right, and not in relation to more general forms. This article, in brief, argues that uncertainty is reduced by the following ideal typical forms: (1) decisions made for others by authority (i.e., a decided order of general principles); (2) valuation, by which certainty emerges as the result of actors ascribing values; and (3) contest, in which the certainty is “fought out.” That is, 2 and 3 are not results of an intended process, but of a mutual adjustment.

These three forms depict the ways in which certainty is achieved by means of judgment in social life as a consequence of individual decisions, judgment devices, and various strategies employed to affect values, such as marketing and political voice, across all domains of social life. They enable us to understand how, and under what conditions, values result from mutual adjustment and when they are the result of design (certainty achieved by decision-making). To have such ideal types available facilitates policy decisions, for example, concerning what type of form is to be used, how the forms come about, and the consequences of the different forms. This raises questions concerning why certain forms are used, the consequences for those involved in the forms that lead to uncertainty, and the consequences for those who are affected by these processes. That they are ideal-types means that they are presented in a pure form, which thus plays down empirical variety and the mixed forms that we observe in real life. Although this is a theoretical article, it provides empirical examples to illustrate the theoretical points.

## Institutionalizing certainty

Uncertainty is diminishing due to conventions and practices that grow out of actors who mutually adjust to one another, e.g., norms, or due to deliberate attempts such as setting laws or implementing standards. Certainty, in other words, can be institutionalized by these two ways. Before turning to the forms of uncertainty reduction, institutionalization of certainty is briefly discussed. The focus is here on one way that is used to institutionalize certainty about what is right, wrong, good, or bad, namely standards, resulting from decisions. Scientific laws and social conventions, practices and norms result from a process of mutual adjustment of actors. Both the deliberately made standards and the unintended emergence of norms are in the existing literature discussed in great detail (Busch 2011, pp. 3–4).

By having law, standards uncertainty is reduced. Law, more specifically natural law, is defined as “any criteria of right judgment in the matter of practice (conduct, action), any standard for assessing options for human conduct as good or bad, right or wrong, desirable or undesirable, decent or unworthy” (Finnis 2002, p. 1). This definition encapsulates the core of what is meant by decision of general principles, which means that the uncertainty is radically diminished already before adjudications, i.e., before judges decide on cases (if it should go that far).

To enable coordination and calculation, thereby reducing uncertainty, standards can, and have been, introduced. Standards refer to the simplification of interaction and coordination and are a result of decisions. When, for example in the field of business, industrial-technical standards were introduced, it became possible to relate to these as facts, and it made business and technical development easier and cheaper (Brunsson, Jacobsson, and associates 2000; Pentzlin 1959). Many goods were standardized to enable transaction of derivatives instead of the actual physical goods, in exchanges (Aspers 2011). To adjust to standards is more a routine than it is decision making (Luhmann 1987, p. 401).

Standards can be used for the purpose of evaluation. Evaluation refers to the act of benchmarking “things,” such as cars, groups, and people, according to a standard. The thing or concern that actors are uncertain about can thus be settled. As an ideal type, no one has to make a decision or ask someone whether whatever is at stake is “good” or “bad”; the standard can be seen as a scale for measuring quality—or whatever is measured—in an “objective” way (Aspers 2009). Evaluation is thus defined as the process by which value is conferred on actors or things, based on a given standard, That is to say, it is institutionalized and independent of individuals’ views or preferences. If it is a purely technical standard this may be obvious, and by technical devices objective evaluations can be made. In the strictest sense, evaluation implies the elimination of judgments. Evaluation, as it were, is independent of who is evaluating and who is evaluated. One may have opinions about how reasonable a standard is, but not about how it is measured. Hence, also when people are involved as evaluators it is “objective” and evaluation is not (supposed to be) based on their “opinions,” “views,” or “preferences” (Busch 2011, pp. 57–58). In reality, however, judgments are included in many evaluations with proclaimed standards.

Based on a standard, it is possible to evaluate things, humans or non-human, directly, according to their properties; for example, depending on their weight or wealth, or indirectly for their deeds, such as being “ethical,” or running fast, or the quality, including their tolerance, of screws made for machines. This means that one can rank them as being “good” or “bad” or being “more” or “less,” according to the standard. But what if there are, for example, no laws—man made or natural laws—that can guide us; how is uncertainty reduced in cases when we are left to our own views or preferences?

## Approaches to uncertainty reduction

To the best of my knowledge, there is no available text that deals explicitly with forms of reducing uncertainty. The sources of uncertainty are many, including lack of decisions and lack of standards, but most research revolves around the issue of value, valuation, and evaluation, with more or less direct connections to uncertainty (cf. Lamont 2012; Zuckerman 2012). Work has been done on valuation, including actual moments of valuation (Berthoin Antal et al. 2015), many of which can be seen as single decisions that together make up the order of worth that is the outcome of valuation processes, as shown by Menger (1999, 2014). Simmel stresses how selection of incumbents for positions is partly stochastic (1923, pp. 183–185). Stark (2009) argues that values are the outcome of dissonance.

There has also been discussion of values and value conflicts. Lamont stresses the existence of matrices of evaluation, and she studies above all practices of evaluation and valuation (Lamont 2009, 2012). There has been work on commensuration (Espeland and Stevens 1998; Fourcade and Healy 2007), namely the process through which several values or entities are reduced to one common denominator. In reality, most forms of valuation imply a combination or commensuration of different values and the exclusion of other values, that is, values that are not part of the process. Vatin introduces the notion of valorization, referring to the manner in which the worth of something increases in processes; he also points out that evaluation and what he calls “valorization” are intertwined in reality (Vatin 2013, p. 32). Much work has been done on the variety of specific values (e.g., Boltanski and Thévenot 2006; Pareto 1935), tools, and strategies involved in processes of valuation and evaluation (cf. Aspers and Beckert 2011; Beckert and Musselin 2013; Karpik 2010; Stark 2009). Values in markets have also been discussed (e.g., Alexius and Hallström 2014).

There is a literature on uncertainty reduction by means of attempts to affect and to control the future (Beckert 2016). Lucien Karpik’s (2010) work is especially relevant to what is discussed here. He addresses the problem of uncertainty concerning which individual product is good; in other words, what non-homogenous offers are of high or low quality. His analysis is presented as standing in opposition to neoclassical economic analysis, in which the market and price differentiation of homogenous offers are at the fore. The tools Karpik presents for assessing quality are called “judgment devices.” The word “device” is rooted in “division”; division, between qualities, or in more general terms, different values, is exactly what these devices enable. These devices are means of separating what is good in markets from what is not good, and they make it easier for actors to make informed choices; they are also "guideposts for individual and collective action" (Karpik 2010, p. 44). Judgment devices reduce the quality uncertainty of products in markets.

There are at least five devices. *Networks* are an actor’s ties through which trustworthy information of what is good flow. It is a form of social capital for adjudicating the quality of products and services in markets. Ties between actors cannot be institutionalized, and they fall outside the analysis of this article. *Cicerones* are critics, that is, experts, and guidebooks that "embody a soft, symbolic form of authority" (Karpik 2010, p. 46). The *Michelin Guide* in the French restaurant market is one such example (Karpik 2000), comparing, selecting, and rating restaurants according to a set of judgment criteria. *Confluences* are techniques that sellers use to form buyers’ decisions, "ranging from territorial location, spatial organization and displays to selling skills" (Karpik 2010, p. 46). In other words, by means of how objects—such as clothes or cars—are presented for sale, where those objects are sold and the style of selling, information is sent out to potential buyers. *Rankings* are the ordering of alternatives: bestseller lists such as those for books or cars, but also the rankings for academic researchers, restaurants, teachers, or wine are instances of this device. These lists are created, for example, by websites or magazines. Credit rating firms, such as Fitch and Moodys are also instances of the ranking device (Rona-Tas and Hiss 2011). The fifth device is *appellations*, which area form of standard. These include labels that inform consumers, in a broad sense, about origin, certifications, brands, and professional titles as institutions of quality. Often a third party makes these appellations legitimate. These devices can exist and be used in various combinations.

Karpik makes an important contribution to our understanding of how uncertainty is reduced. His typology of devices includes empirical categories, some of which are strategies, such as confluences and networks, whereas others are more like public statements of uncertainty reduction, such as rankings. He focuses on a particular type of market good. Forms of uncertainty reduction, however, cover a much larger domain, encompassing all types of things, as well as people, whether in markets or not. The approach taken in the present article looks at some of the basic forms that increase certainty of matters in social life. Most of the cases here refer to objects that are compared with one another, implying that some form of commensuration takes place. Much of Karpik’s work, most clearly rankings, can be integrated into a more general frame of uncertainty reduction, which offers explanations of concrete cases.

The best-known form by which uncertainty is reduced is perhaps the market. The order of worth, manifested in prices of objects, is the outcome of evaluations or valuations carried out by the individuals in the market. However, others have shown (Aspers and Beckert 2011; Vatin 2013) that much of what goes into valuation in markets is the result of non-market valuation processes. Prices in markets result from actors coming together and ordering products. This ordering is of use to those who are actively taking part in the activities, but also—by way of signaling (Spence 2002)—to others. In economics there is also a wide discussion on tournaments, which arguably are about reducing uncertainty, but this has been used primarily to account for wage differences in hierarchies; the higher pay for those with higher rank can be seen as a reward for winning several “competitions” to get promoted (Rosen 1981). In biology the notion “contest” is often used (Fitzpatrick et al. 2012), However, the economic use of tournaments and the biological use of contest are, above all, metaphorical.

Uncertainty is obviously not restricted to markets. When the next president of a club is selected or “the best paper of the year” in a particular section at an American Sociological Association meeting is awarded to an author, it implies a ranking of the candidates. When a group of performers reach agreement on what is a good performance, or when, after voting, the winner of the Eurovision Song Contest is announced, uncertainty is diminished. The announcement of every winner, however, triggers excitement for next year’s contest. In academia we are familiar with evaluation, ranking, and the like, both due to New Public Management and to collegial procedures. As a result of uncertainty reduction, predictability, coordination, and decision-making are facilitated.

## Three forms of uncertainty reduction

There are, I propose, different ways to reduce uncertainty when there is no institutionalized certainty, i.e., lack of standards or other means for objectively knowing and adjudicating what to do and or what will happen if one acts in a certain way. All of these ways increase certainty of matters in social life by sorting out what, for example, is of high quality and has worth, what is to be highly priced—what, in short, is good, and consequently, what is bad. By this differentiation and ordering, whatever things are at stake are positioned in relation to one another in a way that not only makes them different, but also orders them so that some are better than others, so that we can speak of a hierarchy (at least on an ordinal scale).

Forms can exist in domains, and the particular domains over which the resulting ordering has an impact may differ: the FIFA World Cup encompasses all national teams, in contrast to national cups, which order a much smaller part of the social world. Ordering may have large or small implications for social life. There may of course be conflict over values, there are processes for establishing forms, actors may react to what is happening, and much more. Moreover, reduced uncertainty in one domain does not necessarily result in increased uncertainty at a more global level. In many cases the forms reproduce order over time, and in this sense uncertainty is preserved at a certain level; the absence of a form would, in contrast, increase uncertainty. For example, the ranking of academic journals, which is done every year, will not necessarily diminish uncertainty from year to year, but the absence of any ranking would increase uncertainty. In a sense, uncertainty may in some cases be transformed, rather than fundamentally reduced. In other words, the complexity of social life must not diminish to implementation of any of the forms descried here. However, analysis of these implications, which affect observable outcomes, fall outside this article, as does any way to reduce uncertainty that does not directly include human preferences, wants, and interaction among people.^2^ I now turn to the three forms of uncertainty reduction.

### Decision

Decisions can be made to establish general principles that reduce uncertainty by means of institutionalization as discussed above. But uncertainty can be reduced by decisions, without establishing general principle or institutionalized certainty. Decision, as discussed under this heading, represents an adjudication. The uncertainty is reduced for those “deciding” and for those “affected” by the decision. Here the focus is on those affected by others’ decisions (e.g., Brunsson 2007), not on decision theory. All decisions to affect others are attempts to accomplish something, by which one of several alternatives is chosen (Luhmann 1987, p. 399). Decisions made for others by one actor who has authority to determine good and bad is one obvious way of reducing uncertainty, at least until the next decision is made. The authority may arise from a variety of sources (Weber 1978). There must, moreover, be a reference group (Merton 1957) for which the decision supposedly matters to constitute a form. The decision may be made by the chief of a tribe, a king, or a head of a department, or by a governing body deemed legitimate to make decisions for a group of “others.” There is often an expectation on the part of others that a decision is to be made (Luhmann 1987, p. 400). In other words, only if people orient themselves towards and, in a sense, accept a decision will it result in an ordering. Thus, not just anyone making a “decision” on what is good and bad, such as when one decides to have a cup of tea instead of coffee, will directly lead to an ordering of the world. The form of decision refers to case of *decisions* made for others, but not to decisions of general principles.

There are many examples of decisions that attempt to reduce uncertainty. A head of department may decide which faculty members get some of any extra research time that might be at his or her disposal and the coach of a soccer team decides who gets to play in the final. Promotion of potential candidates within a bureaucratic organization after a vacancy may cause uncertainty about who will get the position. This, in turn, may lead to stress, interaction, and attempts at positioning among candidates. A decision by a superior may diminish this uncertainty. Decisions, in their pure form, are unambiguous: if it is decided that A is better than B, and that B is better than C, there is no doubt as long as the decision is clear. Actors then react to the decision and adjust their behavior. This means that the decision has consequences.

Let us look at another example, the case of establishing what is—or to be more accurate, what is seen as—good fashion design. When a few well-known fashion designers take on the role of judge in a recurrent designer competition to award the “designer of the year,” this constitutes a form for valuation.^3^ That the competition is recurrent and that every year established designers enact the role of judge mean that it is institutionalized, but not the outcome. The legitimacy of acting in this role is the joint effect of this institutionalized form and the established actors who are members of the jury. The verdict of this judge—in other words, what is valued in the form—is determined largely by the judge’s status, which is a narrative that is linked to his or her name, and on which status is endowed. As a consequence of his or her verdict and the ranks the competitors obtain, their identities can be ordered in relation to each other. Uncertainty, consequently, diminishes among the competitors and those who have an interest in identifying “good” designers and avoiding those who are not “considered good”.

A decision may diminish uncertainty about the things considered; the same decision may, however, disrupt other things, and consequently—seen in a wider context—increase uncertainty. Frequent and erratic decisions may thus hence increase uncertainty at a more global level. Leaders with the formal authority to decide, but who are deemed to be irrational or unpredictable may cause further uncertainty by their behavior. Their power increases where institutions and standards are few.

### Valuation

A decision requires no external principle of justification; it is enough that the actor making the decision has legitimacy, though such principles of course may bring legitimacy about (Boltanski and Thévenot 2006). The result is a decided order. In other cases the reduced uncertainty is emergent, i.e., resulting from interaction of actors leading to an outcome. Valuation is here defined as the form by which values are ascribed to actors or things based on peoples’ views (preferences). It is thus directly contrasted with evaluation, in which value is given to actors or things based on standards that exist and are in use independent of individuals’ views or preferences; valuation is about differentiation and evaluation is about uniformity. In contrast to a decision that requires only one actor who makes it, valuation is a result of mutual adjustment in a process in which many actors take part.

For valuation, not only do people’s views matter; who these people are matters, too. More precisely, who they are is conceptualized in terms of identity—each has more or less status. This is to say that the “outcome” of the empirically existing instances of the forms, in terms of what is “good,” boils down to peoples’ “emotions,” “preferences,” or, more generally, what they value (cf. Kant 1957, e.g., pp. 198–203). The determination of what is good and bad can be made in discussions among members of a group, or by an “audience” (cf. Bourdieu 1984; White 2002; Zuckerman 1999). In markets, for example, the relationship between those who perform something and their audience “constitute[s] one of the bases for evaluating the producers and their products” (Bourdieu 1993, p. 46; cf. Goffman 1971). The audience, which may be composed of ideal-typical consumers (cf. White 1981), by acknowledging the actors who take part and what they do, endows the actors, or indirectly endows their deeds, with status (Smith Spence 1974). In this way, a rank order of actors making up the social structure emerges. Audiences thereby fulfill a “function” similar to the standard, although they cannot do this with a single act or decision, since they are not a single actor. But an audience, too, provides valuations and an ordering of alternatives that reduces uncertainty concerning what is “good” and “bad” (e.g., “in fashion,” “out of fashion,” and so on). The important difference, to a standard, is that what the audience will say about a “performance” cannot be known in advance. This kind of situation is common in the art world (Beckert and Rössel 2004; Bourdieu 1993; Menger 1999; Plattner 1996; Velthuis 2005), among critics (Bourdieu 1996), and in the markets in these spheres, as well as, generally speaking, in situations characterized by aesthetic values (Podolny 2005, p. 192; Warde 2002), and where there are goods that have been called “singularities” by Lucien Karpik (2010). Fashion (Aspers and Godart 2013) and valuation in its specific markets, such as valuing models (Entwistle 2009; Mears 2011), are perhaps the clearest examples of status forms of valuation, since no standard exists and also what is “beautiful” is subject to fashion. Fashion, for example, cannot be the result of a decision; it is instead the result of valuation (Aspers and Godart 2013).

The form of valuation is made up of the relevant roles—those evaluating and those being evaluated. In this form, it is the institutionalized role structure that orders its environment. In contrast to decision, it is not one single actor who decides; the result is an outcome of many single decisions that jointly and in a process of mutual adjustment result in an outcome. The output is, as in the case of evaluation, numbers or the rank order of whatever is ranked. By ranking or valuing a “thing” an implicit value is created by the actors who are enacting the roles of the form.

Because status orders are made up of actors’ identities and these are more stable than what they give off (for example, produced commodities or verdicts), it is more difficult than in cases based on a standard to know what to do to reshape one’s status (for example, to move up the status ladder). This means that the influence an actor has depends on the position in the social structure. Consequently, actors’ orientations are directed to one another, as indicated by the centrality of gossip (cf. White 1993, p. 167), because there is no standard to which those who ascribe value and those who are ascribed values can orient themselves.

In academia, we are familiar with the review process, of potential publications, proposals, positions, and promotions. This may be presented as a standard of quality, and, though reviewers tend to agree, it indeed matters who is reviewing, for example, proposals (Roumbanis 2017). If it did not matter, we would, as authors, be able to predict whether a paper is good enough to be published even before submitting it to the journal. Furthermore, there would, in principle, be no rejections, because authors would easily know in advance whether the paper is good enough to be published. The high rejection rates of the top journals, which arguably uphold the scientific quality standard, indicate the uncertainty of success (cf. Menger 2014).^4^

### Contest

Contest is a direct way to settle uncertainty between those taking part, a result that radiates to all those with an interest in the contest. Combat, duel (Ciklamini 1963), or war, by which uncertainty about strength of the parties is settled, are noticeable examples since life is at stake.^5^ The outcome of the struggle is uncertain prior to the contest. The notions of struggle and conflict are correlates and above all discussed in terms of their forms and consequences by Georg Simmel (cf. Simmel’s notion of *“Kampf”* (struggle); Simmel 1923, pp. 186ff). He was not, however, focusing on the issue of uncertainty.

A contest may start as a negotiation and end with violence, such as combat, or vice versa; in both cases actors are in opposition. Combat as a way of reducing uncertainty is, however, intransitive; it only settles the uncertainty between those combatting. For example, if boxer A won against B at a certain point in time and B around the same time won against C, it does not follow that A would definitely win against C.

In a contest there are rules determining what is to be done to achieve what is good and not so good—rankings, positions, and so on. Only those battling directly affect the outcome. Others’ views, ideas, and arguments do not matter in the ideal-typical world. In reality, of course, things are less clear. A typical example is boxing (Øygarden 2000). Before the match, uncertainty about the outcome may be high, and the stakes for those involved are extremely high. Uncertainty is both the reason for the match and what may cause the hype with speculation, betting, verbal assaults, and the enormous emotional charge that is often recognized between boxers in a title bout and in the audience. That one boxer has the great majority of supporters shouting his name may affect how he is doing, but no matter how much they shout, if he is knocked out he has lost. It is indisputably all over. The shouting crowd and many other things, too, will affect the judges and the referee. Even in cases in which there is a knock out or if the judges decide on the outcome using points, the notion of contest still makes sense; the interaction is direct, and the outcome follows from the contest.^6^ The contest, though it may work itself out in many ways, diminishes uncertainty; one participant loses; the one still standing after a boxing match is the winner. Provided that the rules have been followed, the result of the ordering cannot be undone and the outcome is^7^ publicly known. Also combat, duel, and war will make this clear.

There are several empirical examples by which outcomes of contests result in reduced uncertainty. E-sport, or competitive gaming, tennis matches, and boxing matches are examples that generate order resulting in rankings, prizes, or the like. In this way uncertainty about who is good and not so good is reduced, or even settled, at least temporarily. Games have a temporal structure; as soon as one game ends, the focus can shift to the next one. Associations capitalize on this insight to run leagues and not just single matches.^8^

Several contests that are tied together in a system of rules represent a tournament or a sequence of connected contests. There are various forms of tournaments, such as the round-robin (everyone involved meets at least once), such as the soccer Premier League in the United Kingdom, or elimination tournaments in which only the winner stays in the competition, such as the US Open in tennis. In tournaments, the players meet and “fight” directly with one another to settle, normally according to rules, what (or who) is (good) and what is not (so good). The result is order. The World Cup in soccer is a contest that in the end produces certainty about quality of the teams: the cup and gold medals go to the winning team, the silver to the team that comes second, and so on. Moreover, a formal ranking list with all member nations is presented by FIFA. This ranking is not the result of a decision, not an evaluation, not a valuation, but results ultimately from the scores achieved on the pitch.

Contest also includes the idea of a combat, or even war, as a state of violent conflict with weapon by means of several battles over time between groups (states) (Janssen et al. 1987, p. 703), including a war of “all against all.” Some computer games use a multiplayer online combat arena in which different players or teams of players are involved in the combat. The combat usually ends when the opponents’ bases have been destroyed or when there is one part still standing. The teams can be ordered according to their scores, or one can create tournaments of different types to let more than two actors enter a combat.

## Future research

This article presents three ideal-typical forms of uncertainty reduction. One form, decision, represents a decided order, and the other two forms result from mutual adjustment. As theoretical forms, they cannot be reduced to one another. Generally speaking, hybrid forms of ideal types exist in real life, as indicated above. One may, for example, think that the verbal duels in rap, known as “rap battles,” are pure instances of what I have called “contest” in this article, but the language leads us astray. Although there are rules, the ultimate outcome of a contest—who wins—is a result of the onlookers’ response: “The participant who is able to get louder and longer reactions from onlookers wins the verbal duel” (Lee 2009, p. 581). In contrast to boxing, in which a boxer may have the entire crowd of onlookers against him, but still win, the rap battle is much more a form of valuation, in which the onlookers’ preferences diminish the initial uncertainty concerning who is the best rapper of the two.

This article cannot cover all relevant aspects or the consequences that follow from uncertainty reduction. Let me briefly mention six additional issues that might be addressed in relation to a discussion of these forms. The first concerns material objects. I have been discussing almost exclusively about actors—mainly humans—in this article. Uncertainty reduction refers also to material objects, as well as organizations that gain meaning in these social processes. Only actors can make decisions and be accountable for them. In contests, actors take part, directly, or indirectly; for example, when cocks are fighting, as described by Geertz (1973, pp. 412–453). Objects, such as antiquities, cars, or model trains, are valued by people in relation to other objects, such as horses and chairs, but they are ordered in relation to other antiquities, cars, and real trains.

Objects do not have reflexive capacity, and therefore cannot directly operate as an audience, for example in cases of valuation. Material objects can, however, be part of the ranking process of humans or other objects. When athletes compete, for example, by running the 110-m hurdles, the time is measured by a clock connected to other technical devices, including a camera, track, hurdles, and a computer. Standards or “technologies” and thus “equipment” can replace objective judges of standards (a “quality”) to “test and measure the goods” (Callon et al. 2002, p. 199), which is to say that man can be a function of, and thus ordered by technology (Heidegger 1957, p. 271). However, human beings can also become integrated in technological production systems. Timber trading is a good example of how this can be done. Timber is traded in different quality segments, and in this “pure” standard market, the evaluation of the quality classes of timber is made by humans (Aspers 2013). Generally speaking, we should address the relations among the taken for granted lifeworld, codified institutions, and the “outcome” of the forms of uncertainty reduction (Husserl 1970).

The second issue that we can discuss in more detail is how these ideal-typical forms are fashioned and how they disappear. These forms, for example, may be the result of individual decisions; either as a result of mutual adjustment (“spontaneous order”) or a decided order. The forms discussed may last for a very short period, but most, such as markets, are fairly stable (Burt 1988). The forms may also gradually turn into one another. Weber (1978, pp. 770–771) describes how combat is gradually transformed into the “thing” (the old Scandinavian term for a place to settle disputes among free men without weapons), and gradually come to rest on formal law (evaluation/valuation) (cf. Elias 1969). How do forms turn into other forms, and does it matter?

A third issue concerns the domain of the forms. Within the economy, much uncertainty is reduced by markets. Markets order alternatives and provide values for them. Firms gain identities in producer markets (White 1981) as a result of valuation by customers. In other markets, typically with homogenous products—such as the trading of stocks on an exchange (Walras 1954)—evaluation of price appears to dominate; the stocks themselves are identical and “given.” Decisions on value may occur in some cases; this is typical of socialist economies (Gronow 2003). Direct physical “contests” are today unusual in the market economy, but negotiations, a peaceful form of “contest,” are central. We cannot, therefore, rule out any form in any sphere of life. To what extent do the forms format the domains?

A fourth issue is the important fact that these forms provide not only certainty and order “within” a domain. Perhaps just as important, but less discernible, is their gatekeeping function. Only those who are considered with regard to, or included in, the form are serious contenders for being seen as “good.” To be a boxer one has to have been in the ring. To be an academic one needs an advanced degree, and to be an author one must be published. Many try to take part in different activities, but are refused entry; this is particularly true of those trying to publish their first novel, who often are involved in a process with limited chances of success and requires much effort and commitment from those trying (Furst 2017). Which forms are “efficient” for gatekeeping and which are open for entry?

A fifth issue is the existential concern that often follows from judgment concerning one’s body, performances, deeds, or way of life. Regardless of whether one wants to take part in a form or not, one has to relate to the “verdict”; simply because what people are and do is crucial for their identities (Mears and Finlay 2005; White 2008). Thus, actors may be affected by what others do; they may be “pulled in” to be evaluated, valued, or even challenged in contests, even against their will. This exposure causes reactions, leading to actions and reactions (Espeland and Sauder 2007; Furst 2017). But what existential consequences can we observe?

A sixth issue concerns the more general societal level. In this article the theme is uncertainty reduction. But uncertainty is also a trigger for commercial activity, creative work, and much more (Menger 2014; Beckert 2016). Uncertainty is often produced, for example to make profit, and the large number of television shows that are based on competitions and eliminations, sets the scene for those who profit from “uncertainty.” Many of the various television shows use the forms described in this article to combine the effects of enhanced uncertainty, before it is eventually diminished when the winner eventually emerges. It is important to realize that the forms themselves are not inherently oriented towards the betterment of the world. A completely certain world is not imaginable. What do the attempts of uncertainty reduction lead to?

## Conclusion

Fundamentally, this article addresses uncertainty about values; ultimately “good” and “bad”. Uncertainty is persistent, and even uncertainty reduction itself is an uncertain endeavor (Knight 1921, pp. 347–348); that is to say that uncertainty is a condition of human life. Over time, the types of uncertainty we face have changed. By institutionalization, by setting laws, and by producing scientific knowledge decreased some uncertainties have decreased, while others have been created. Most of us can be fairly certain that there will be food on the table tomorrow, but perhaps not be certain that the globe can accommodate our pollution. Uncertainty is fundamentally about not knowing the future. Institutionalized certainty increases predictability and increases our chances of controlling the future.

Based on the idea of forms as means of uncertainty reduction, this article makes two contributions. The first is the presentation of the forms: decision, valuation, and contest. The findings of the article adds to the ongoing discussion of valuation and evaluation, partly by clarifying the role of uncertainty and how there are different forms by means of which this uncertainty may be reduced.

The more theoretical contribution of this article offers tools for addressing and understanding the activities, processes, and trends that point towards the increased role of competitions, rankings, auditing, and evaluations in social life. The process of marketization (Braudel 1992; Polanyi 2001), which indeed has accelerated over recent decades, increases the number of areas of uncertainty. Furthermore, in some markets there are no standards to adjudicate between the different offers, typically status markets, in other markets the presence of standards enable just this type of comparison of “quality” of the offers, of which type the perfect market with homogenous products is the extreme instance. Moreover, the increased role of auditing (Power 1997), evaluation (Dahler-Larsen 2011), and standardization (Brunsson, Jacobsson, and associates 2000; Busch 2011), including the construction of tools that can be used to evaluate and benchmark risks (Power 2007), should be seen as ways in which actors try to control and bring more certainty to the world, often by trying to impose it using standards, and thereby diminishing the role of valuation. Some of these standards and evaluations are global, which is notable in relation to global markets, but also non-governmental organizations and standard settings, in which firms, organizations, and even states are evaluated according to the same standard, such as accounting standards (Djelic and Quack 2007, pp. 174–181). The forms of uncertainty reduction do not make the world “certain” in any sense; these are mere attempts to, as we have seen, both create and reduce uncertainties.

## Footnotes

1 There are of course always ways of questioning institutionalized knowledge, but the point is that the decision is made on the existing evidence. Uncertainty, moreover, is not ontological but epistemic and implies, for example, that though one knows that there is a restaurant on the block (an “ontological” statement), one does not yet know whether it is good or not in relation to other restaurants (an epistemic statement).

2 Randomization is one alternative, but it is hard to see how it reduces uncertainty, it merely shifts the basis of uncertainty. Aristotle (2000, e.g., pp. 250, 252), for example, suggested that the lottery offers a way of addressing the uncertainty of elections to avoid. A lottery distributes whatever is at stake based on probabilities, but it does not in any relevant way represent interdependent interaction.

3 For an example, see http://designaward.hm.com/ (accessed December 07, 2017).

4 The review process, though it to some extent resembles the form of evaluation, also resembles the form of valuation, both in academic review processes (Lamont 2009) and in publication of novels (Furst 2017).

5 Elias points out that the war and fighting was normal among knights in the Middle ages, but this violent way of interaction gradually gave way to more civilized interactions (1969, pp. 263–301).

6 Corrupt judges who make their decision regardless of course do exist. And in contemporary military conflicts it is the international audience, not least United Nation’s Security Council, which decides on outcomes. But with the strong external parties making decisions, we can no longer speak of a strong, or “pure,” form.

7 In even more violent games, such as MMA this is even more distinct, since it less frequently comes to a draw.

8 Football was originally a single game activity; only later were leagues (and tournaments) organized (Döllinger n.d.).
